# Supplementary material for: Neurogliaform cortical interneurons derive from cells in the preoptic area
Source: eLife. 2018 Mar 20;7:e32017. doi: 10.7554/eLife.32017 (PMC5860868; doi:10.7554/eLife.32017)
Supplement: Supplementary file 1. [file elife-32017-supp1.docx]

### Supplementary Table 1: Cell count and number of brains used for quantifications

| **Age** | **Type of tissue** | **Brains** | **Cells counted as the 100%** | **Cells** | **Colocalisation analysed** | **Shown in** |
| --- | --- | --- | --- | --- | --- | --- |
| E14.5 | *Hmx3*-tdTOM^+^;*Htr3a*-GFP^+^ | n=3 | *Htr3a*-GFP | n=2030 (POA)  n=1970 (overlap zone) | *Hmx3*-tdTOM | Figure 1C |
|  |  |  | *Hmx3*-tdTOM | n=3469 (POA)  n=1986 (overlap zone) | *Htr3a*-GFP |  |
|  |  |  | *Hmx3*-tdTOM; *Htr3a*-GFP | n=1675 | NR2F2/SP8 | Figure 1E |
|  |  |  |  | n=463 | PROX1 |  |
|  |  |  |  | n=1146 | NKX2.1 | Figure 1F |
|  |  | n=2 | *Hmx3*-tdTOM; *Htr3a*-GFP | n=120 | *Htr3a* mRNA | Figu­­re 1D |
|  |  |  | *Hmx3*-tdTOM | n=158 | *Htr3a* mRNA |  |
|  |  |  | *Htr3a*-GFP | n=30 | *Htr3a* mRNA |  |
| P5 | *Hmx3*-tdTOM^+^;*Htr3a*-GFP^+^ | n=2 | *Htr3a*-GFP | n=1118 | *Hmx3*-tdTOM | Figure 2B  Figure 3C |
|  | *Dbx1*-tdTOM^+^;*Htr3a*-GFP^+^ | n=3 | *Htr3a*-GFP | n=5741 | *Dbx1*-tdTOM | Figure 3C |
| P9 | *Hmx3*-tdTOM^+^;*Htr3a*-GFP^+^ | n=2 | *Htr3a*-GFP | n=878 | *Hmx3*-tdTOM | Figure 2B |
| P21 | *Dbx1*-tdTOM^+^;*Htr3a*-GFP^+^ | n=3 | *Htr3a*-GFP | n=2551 | *Dbx1*-tdTOM | Figure 3C |
|  |  |  | *Dbx1*-tdTOM | n=266 | *Htr3a*-GFP/PROX1/SOX6 | Figure 3D |
|  | *Hmx3*-tdTOM^+^;*Htr3a*-GFP^+^ | n=3 | *Htr3a*-GFP | n=1029 | *Hmx3*-tdTOM | Figure 2B |
|  |  |  | *Hmx3*-tdTOM | n=798 | *Htr3a*-GFP | Not shown |
|  |  | n=2 | *Hmx3*-tdTOM | n=453 | NeuN | Figure 2E |
|  |  |  | *Hmx3*-tdTOM; *Htr3a*-GFP | n=512 | NeuN |  |
|  |  | n=3 | *Htr3a*-GFP | n=1593 | PROX1/SOX6 | Figure 4B |
|  |  |  |  | n=1612 | NR2F2/SP8 | Figure 4D |
|  |  |  | *Hmx3*-tdTOM; *Htr3a*-GFP | n=308 | PROX1/SOX6 | Figure 4B |
|  |  |  |  | n=337 | NR2F2/SP8 | Figure 4D |
|  |  |  | *Hmx3*-tdTOM; *Htr3a*-GFP | n=166 | Reelin | Figure 5C |
|  |  |  |  | n=171 | NPY | Figure 5E |
|  |  |  | *Htr3a*-GFP/Reelin | n=302 | *Hmx3*-tdTOM | Figure 5D |
|  |  |  | *Htr3a*-GFP/NPY | n=248 | *Hmx3*-tdTOM | Figure 5F |
|  |  | n=2 | *Hmx3*-tdTOM; *Htr3a*-GFP | n=1301 | Reelin/NPY | Figure 5G |
|  |  |  |  | n=78 | VIP | Not shown |
| P30 | *Hmx3*-tdTOM^+^;*Htr3a*-GFP | n=2 | *Hmx3*-tdTOM; *Htr3a*-GFP | n=80 | *Car4* mRNA | Figure 6B  Figure 6 Supplement 3B, D |
|  |  |  | *Htr3a*-GFP | n=86 |  |  |
